# Secondary Hemophagocytic Lymphohistiocytosis Mimicking Sepsis in Adults: A Case Series

**DOI:** 10.3390/reports9040329

**Published:** 2026-09-28

**Authors:** Damián Ochoa Guette, Jennifer Patricia Vargas Gomez, Breallan De Jesús Romero Pájaro, Rafael Tous Bertel, Amilkar Almanza Hurtado

**Affiliations:** 1 Faculty of Health Sciences, Department of Internal Medicine, Universidad Libre Barranquilla Campus, Barranquilla 08000, Colombia; jenvargasg@gmail.com (J.P.V.G.); breallan-romerop@unilibre.edu.co (B.D.J.R.P.); 2 Faculty of Medicine, Department of Critical Care, Universidad del Sinú Cartagena Campus, Av. El Bosque, Transversal 54 No. 30-453, Santillana, Cartagena de Indias 130015, Colombia; rafaeltous8@gmail.com; 3 Faculty of Health Sciences, Department of Critical Care, Universidad de Cartagena Campus, Av. El Bosque, Transversal 54 No. 30-453, Santillana, Cartagena de Indias 130015, Colombia; dr.amilkar.almanza@gmail.com

**Keywords:** hemophagocytic lymphohistiocytosis, hyperinflammation, cytokine storm, sepsis, hyperferritinemia, histoplasmosis, visceral leishmaniasis, acute myeloid leukemia

## Abstract

**Background and Clinical Significance:** Secondary hemophagocytic lymphohistiocytosis (HLH) is a life-threatening hyperinflammatory syndrome that can closely resemble sepsis in adults, potentially delaying recognition and treatment. Because infection may trigger or coexist with HLH, persistent or disproportionate inflammation despite appropriate antimicrobial therapy should prompt reassessment for concomitant HLH and investigation of the underlying trigger; **Case Presentation:** We describe three adults initially managed for presumed sepsis or severe infection at a referral hospital in Colombia. All developed persistent fever, cytopenias, organomegaly, and marked hyperferritinemia. HScore values were 222, 246, and 248, respectively; the score in Case 3 was calculated retrospectively from available pretreatment data. Bone marrow hemophagocytosis was demonstrated in Cases 1 and 2 but not in Case 3. The underlying triggers were acute myeloid leukemia, disseminated histoplasmosis in the setting of advanced HIV infection, and visceral leishmaniasis. The malignancy-associated case had an unfavorable outcome. Both infection-associated cases improved after pathogen-directed therapy combined with corticosteroids to control hyperinflammation, without escalation to an etoposide-based regimen; **Conclusions**: Sepsis and secondary HLH should not be regarded as mutually exclusive diagnoses. Persistent or disproportionate inflammation accompanied by progressive multilineage cytopenias, splenomegaly, markedly elevated or rising ferritin levels, hypertriglyceridemia, and/or decreasing fibrinogen should prompt early HScore calculation, assessment of HLH-2004 criteria, and concurrent investigation for an underlying trigger. Recognition of the clinical pattern and individualized treatment of both the trigger and hyperinflammatory state may facilitate timely management.

## 1. Introduction and Clinical Significance

Hemophagocytic lymphohistiocytosis (HLH) is a severe, life-threatening hyperinflammatory syndrome characterized by uncontrolled activation of cytotoxic lymphocytes and macrophages, excessive cytokine release, tissue injury, and potentially fatal multiorgan dysfunction. In adults, HLH is most often secondary to hematologic malignancy, infection, autoimmune or autoinflammatory disease, or other states of immune dysregulation. It is increasingly recognized as part of the broader spectrum of cytokine storm syndromes [1,2].

Recognition of secondary HLH in adults is particularly challenging because its clinical presentation can closely resemble sepsis. Fever, cytopenias, coagulopathy, hepatic dysfunction, organomegaly, hyperferritinemia, and multiorgan failure may occur in both conditions, while infection may itself trigger or coexist with HLH. Consequently, a binary diagnostic approach of “sepsis versus HLH” may delay recognition of concomitant hyperinflammation. Persistent or disproportionate inflammation despite appropriate antimicrobial therapy should therefore prompt parallel reassessment for HLH and investigation of its underlying trigger [3,4,5].

The HLH-2004 criteria and the HScore are the most commonly used diagnostic frameworks in adults, although neither was specifically developed for all critically ill adult populations. HLH-2004 was originally developed for pediatric familial HLH, whereas the HScore was derived in adults with suspected reactive hemophagocytic syndrome. Moreover, diagnostic tests such as soluble CD25 and natural killer (NK) cell function may not be readily available in many centers. Diagnosis in adults therefore requires integration of the clinical course, serial laboratory trends, bone marrow findings when available, and systematic evaluation for alternative or coexisting causes of hyperinflammation [4,6,7,8,9,10].

Hematologic malignancies are major triggers of secondary HLH in adults and are associated with poor outcomes. Intracellular infections are also important triggers, particularly in endemic settings and among immunocompromised patients. Disseminated histoplasmosis and visceral leishmaniasis may cause prolonged fever, profound cytopenias, hepatosplenomegaly, and marked hyperferritinemia, thereby mimicking both sepsis and hematologic malignancy [11,12,13].

Against this background, we report three adult cases of secondary HLH initially managed as presumed sepsis or severe infection at a referral hospital in Colombia. The cases illustrate malignancy- and infection-associated HLH, the limitations of relying on individual diagnostic findings, and the importance of considering competing and coexisting diagnoses in parallel. They also highlight practical clinical features that may prompt earlier recognition of HLH and emphasize the need for timely identification and treatment of the underlying trigger.

## 2. Case Presentation

### 2.1. Case 1

A 54-year-old man with end-stage kidney disease requiring hemodialysis, hypertension, and hypothyroidism was admitted with persistent fever, dry cough, low back pain, constitutional symptoms, and pancytopenia. The initial clinical impression was high-risk febrile neutropenia, and broad-spectrum antimicrobial therapy was initiated. Blood cultures grew *Streptococcus gallolyticus*, and antibiotic therapy was subsequently adjusted according to antimicrobial susceptibility testing. Despite targeted treatment, the patient remained febrile and developed progressive clinical deterioration with persistent cytopenias.

Further evaluation showed marked hyperferritinemia (8975 ng/mL), hypertriglyceridemia (789 mg/dL), and splenomegaly, raising concern for a concomitant hyperinflammatory syndrome rather than infection alone. The patient fulfilled 5 of 8 HLH-2004 criteria and had an HScore of 222, indicating a high probability of secondary HLH. Bone marrow aspirate demonstrated hemophagocytosis (Figure 1A,B), while flow cytometry identified 22.8% myeloblasts with immunophenotypic features consistent with acute myeloid leukemia (AML-M2), including aberrant CD7 and CD56 expression.

The overall clinical picture was interpreted as malignancy-associated HLH in the setting of concomitant *S. gallolyticus* bacteremia. Persistent fever and cytopenias despite targeted antimicrobial therapy, together with marked hyperferritinemia, hypertriglyceridemia, splenomegaly, bone marrow hemophagocytosis, fulfillment of 5 of 8 HLH-2004 criteria, and an HScore of 222 supported HLH rather than sepsis alone as the explanation for the persistent hyperinflammatory state. Fibrinogen was elevated (593 mg/dL), rather than decreased, at the time of HLH assessment.

Given the patient’s end-stage kidney disease, poor functional status, newly diagnosed AML, and palliative treatment goals, azacitidine was selected as malignancy-directed therapy, together with supportive care and treatment of the concomitant infection. No etoposide-based HLH regimen was documented in the available clinical record. The patient’s subsequent clinical course was unfavorable.

### 2.2. Case 2

A 34-year-old man with newly diagnosed advanced HIV infection (stage C3) and severe immunosuppression (CD4 count, 38 cells/mm^3^) was admitted with prolonged fever, weight loss, chronic diarrhea, jaundice, and progressive dyspnea. The initial diagnostic evaluation focused on opportunistic infections, particularly after thoracic imaging revealed a right pulmonary mass with mediastinal extension. During hospitalization, the patient developed diffuse pulmonary infiltrates and hypoxemia, prompting empiric treatment for *Pneumocystis jirovecii* pneumonia.

Despite broad antimicrobial therapy, the patient had persistent systemic inflammation and progressive clinical deterioration, accompanied by hepatosplenomegaly, bicytopenia, hyperferritinemia (3551 ng/mL), and hypertriglyceridemia. Potential alternative or contributing explanations for the cytopenias and inflammatory abnormalities included HIV-related bone marrow suppression, opportunistic infection, viral infection, and hepatic dysfunction. However, the combination of these findings with bone marrow hemophagocytosis (Figure 2A,B), fulfillment of 7 of 8 HLH-2004 criteria, and an HScore of 246 strongly supported secondary HLH rather than attribution of the clinical picture to any single competing diagnosis.

Etiologic investigation identified disseminated *Histoplasma capsulatum* infection, supported by a positive urinary antigen test and histopathologic demonstration of intracellular fungal organisms in pulmonary and mediastinal tissue samples. CMV and Epstein–Barr virus serologic markers were also detected; however, disseminated histoplasmosis was considered the principal trigger of the hyperinflammatory syndrome.

Treatment consisted of liposomal amphotericin B for disseminated histoplasmosis and dexamethasone (4 mg intravenously every 12 h) to control HLH-associated hyperinflammation. Antiretroviral therapy was subsequently initiated after central nervous system infection had been excluded. Because the patient showed progressive clinical and hematologic improvement with pathogen-directed antifungal therapy plus corticosteroids, escalation to an etoposide-based regimen was not pursued. The patient continued to improve and was discharged with outpatient follow-up.

### 2.3. Case 3

A 28-year-old previously healthy man was admitted with an approximately two-month history of fever, weight loss, malaise, and progressive pancytopenia. Abdominal imaging demonstrated marked hepatosplenomegaly. He had previously received treatment for presumed tuberculosis; however, persistent fever and cytopenias, together with the absence of thoracic imaging findings supporting tuberculosis, prompted diagnostic reassessment. Bone marrow polymerase chain reaction (PCR) testing for *Mycobacterium tuberculosis* was negative. An initially reactive HIV serologic test was also considered discordant because HIV RNA was undetectable in the absence of antiretroviral therapy. Given the combination of pancytopenia, hepatosplenomegaly, and elevated lactate dehydrogenase, a lymphoproliferative or other hematologic disorder was also investigated.

Further evaluation showed marked hyperferritinemia (8856 ng/mL), hypertriglyceridemia (230 mg/dL), persistent pancytopenia, fever above 38.5 °C, and hepatosplenomegaly. Bone marrow aspirate demonstrated trilineage hematopoiesis with erythroid expansion but no hemophagocytosis. Flow cytometry showed no evidence of neoplastic infiltration, and conventional cytogenetic analysis demonstrated a normal 46,XY karyotype. Antinuclear antibody (ANA) and anti-double-stranded DNA (anti-dsDNA) testing were negative, providing no evidence to support an autoimmune trigger.

The HScore was calculated retrospectively using available pretreatment values obtained during the diagnostic work-up and assigning no points for bone marrow hemophagocytosis. The resulting score was 248, indicating a very high probability of reactive HLH. The patient initially fulfilled four HLH-2004 criteria, fever, splenomegaly, cytopenias, and hyperferritinemia, and subsequently fulfilled a fifth criterion when fibrinogen decreased to 1.48 g/L during hospitalization. Thus, the absence of bone marrow hemophagocytosis did not preclude the diagnosis of secondary HLH in the context of the overall clinical and laboratory findings.

Visceral leishmaniasis was investigated because of relevant epidemiologic exposure and a compatible reticuloendothelial syndrome. Bone marrow PCR for *Leishmania* spp. was negative; however, a rapid diagnostic test was positive, and indirect immunofluorescence performed by the Colombian National Institute of Health was reactive at a titer of 1:256 (Figure 3A,B). Taken together with the clinical and epidemiologic findings, these results supported visceral leishmaniasis as the most likely infectious trigger of secondary HLH.

Treatment consisted of liposomal amphotericin B together with corticosteroids to control hyperinflammation. Given the patient’s hemodynamic stability and progressive hematologic improvement with pathogen-directed therapy plus corticosteroids, etoposide was not added. The patient subsequently showed clinical improvement and hematologic recovery.

The main demographic, clinical, laboratory, and outcome characteristics of the patients included in this series are summarized in Table 1.

## 3. Discussion

Secondary HLH in adults remains a major diagnostic challenge because its clinical presentation substantially overlaps with that of sepsis. Machowicz et al. emphasized that fever, organ dysfunction, cytopenias, and hyperinflammation may occur in both syndromes and that infection may trigger or coexist with HLH [5]. This overlap was also demonstrated by Liedgens et al., who reported that critically ill patients with cancer and marked hyperferritinemia frequently fulfilled criteria for septic shock and, in a substantial subset, HLH-2004 criteria [14]. Our cases reinforce the concept that sepsis and HLH should not be regarded as mutually exclusive diagnoses. Infection was documented or strongly suspected in all three patients, while persistent or disproportionate hyperinflammation prompted evaluation for concomitant HLH.

The pathobiology of HLH involves impaired or overwhelmed cytotoxic lymphocyte function, persistent antigenic stimulation, macrophage activation, and excessive cytokine signaling [1,2]. Sepsis activates many of the same inflammatory pathways, which helps explain why no single laboratory marker can reliably distinguish the two syndromes. Ferritin is particularly useful as a signal to reassess the diagnosis, but marked hyperferritinemia is not specific for HLH in adults and may also occur in severe infection, hepatic injury, renal dysfunction, and hematologic malignancy [14,15]. Similarly, C-reactive protein (CRP) and procalcitonin (PCT) are useful biomarkers in patients with suspected infection or sepsis, with PCT generally more closely associated with bacterial infection and organ dysfunction; however, neither biomarker excludes concomitant HLH [16]. In clinical practice, markedly elevated or rapidly rising ferritin levels accompanied by progressive multilineage cytopenias, splenomegaly, decreasing fibrinogen and/or increasing triglyceride levels, and failure to improve as expected with appropriate antimicrobial therapy should prompt HScore calculation and formal reassessment for HLH [5,10]. Table 2 summarizes the principal overlapping and potentially discriminating features of HLH and sepsis while emphasizing the limited specificity of individual findings.

The diagnosis of HLH in adults requires integration of clinical, laboratory, and histopathologic findings. The HLH-2004 criteria are widely used but were originally developed for pediatric familial HLH, whereas the HScore provides a probability estimate for reactive HLH in adults [7,9,10]. In Case 3, an HScore of 248 was calculated retrospectively using available pretreatment values, with no points assigned for bone marrow hemophagocytosis. Importantly, bone marrow examination in this patient did not demonstrate hemophagocytosis. This finding illustrates a central diagnostic principle: hemophagocytosis supports the diagnosis when present but is neither sufficiently sensitive nor specific to establish or exclude HLH [5,6,7,8].

Patient-specific HLH-2004 and HScore results are summarized in Table 1 and Table 3 presents a practical red-flag pathway for adults with presumed sepsis who fail to improve, integrating continued sepsis management with reassessment of the inflammatory trajectory, estimation of HLH probability, identification of the underlying trigger, and individualized treatment.

Bone marrow examination remained valuable in this series because it contributed both to the assessment of HLH and to the identification or exclusion of potential triggers. Hemophagocytosis was demonstrated in Cases 1 and 2 but not in Case 3. Bone marrow evaluation also identified AML in Case 1 and helped exclude a clonal hematologic process in Case 3. Therefore, bone marrow examination should be used to investigate both HLH and its potential underlying cause, without delaying clinical assessment while awaiting demonstration of hemophagocytosis [5,6,7,8].

The triggers identified in this series reflect the heterogeneity of secondary HLH in adults. Case 1 represents malignancy-associated HLH in the setting of AML, a subgroup associated with particularly poor outcomes and in which control of the underlying malignancy is central to management [11]. In this patient, severe comorbidity, poor functional status, and palliative treatment goals influenced the decision to use azacitidine rather than intensive AML therapy or an etoposide-based HLH regimen.

Cases 2 and 3 were associated with disseminated histoplasmosis and visceral leishmaniasis, respectively. Both are intracellular infections capable of producing sustained macrophage activation, cytopenias, hepatosplenomegaly, and marked inflammatory abnormalities. Disseminated histoplasmosis is a recognized cause of secondary HLH in immunocompromised patients, particularly those with advanced HIV infection [12]. Visceral leishmaniasis may produce a similar reticuloendothelial phenotype and should be considered in patients with compatible epidemiologic exposure [13]. The diagnostic discordance in Case 3’s negative bone marrow PCR for *Leishmania* spp. despite positive rapid testing and reactive serology further illustrates the importance of integrating microbiologic findings with clinical context, epidemiologic probability, and treatment response rather than relying on a single diagnostic assay.

Management of secondary HLH in adults should address both the hyperinflammatory state and the underlying trigger, while avoiding the automatic application of pediatric HLH protocols to every adult patient. Current adult recommendations support individualized treatment according to the underlying trigger, disease severity, organ dysfunction, comorbidities, and response to initial therapy [10]. In Case 2, disseminated histoplasmosis was treated with liposomal amphotericin B together with dexamethasone (4 mg intravenously every 12 h), with clinical and hematologic improvement without escalation to etoposide. In Case 3, treatment with liposomal amphotericin B and corticosteroids was followed by clinical and hematologic recovery, and etoposide was likewise not added. In Case 1, no etoposide-based regimen was documented; treatment was directed toward AML with palliative azacitidine in the context of severe comorbidity and poor functional status. Collectively, these cases illustrate that the intensity of HLH-directed therapy in adults should be individualized according to the clinical context and treatment response rather than determined solely by fulfillment of diagnostic criteria.

This report has several limitations. First, it is a retrospective series of three patients with heterogeneous triggers, limiting generalizability and precluding causal inference. Second, advanced immunologic testing, including soluble CD25 and NK-cell functional assays, was not consistently available. Third, the HScore for Case 3 was calculated retrospectively using values obtained during the diagnostic work-up rather than prospectively at presentation. Finally, microbiologic evidence was heterogeneous, particularly in Case 3, in whom bone marrow PCR for *Leishmania* spp. was negative despite positive rapid testing and reactive serology. Nevertheless, these cases provide a clinically relevant illustration of the diagnostic overlap between sepsis and secondary HLH and underscore the importance of repeated reassessment when the inflammatory trajectory is disproportionate to the presumed infection.

## 4. Conclusions

Secondary HLH should be considered in adults with presumed sepsis when fever and systemic inflammation persist or worsen despite appropriate antimicrobial therapy, particularly in the presence of progressive multilineage cytopenias, splenomegaly, markedly elevated or rising ferritin levels, hypertriglyceridemia, and/or decreasing fibrinogen levels. Sepsis and HLH may coexist; therefore, evaluation for HLH should proceed in parallel with continued investigation and treatment of infection. Early HScore calculation, systematic assessment of HLH-2004 criteria, and investigation of the underlying trigger may facilitate timely recognition. Treatment should address both the trigger and the hyperinflammatory state and should be individualized according to disease severity, comorbidities, organ dysfunction, and response to initial therapy.

## Figures and Tables

**Figure 1:**
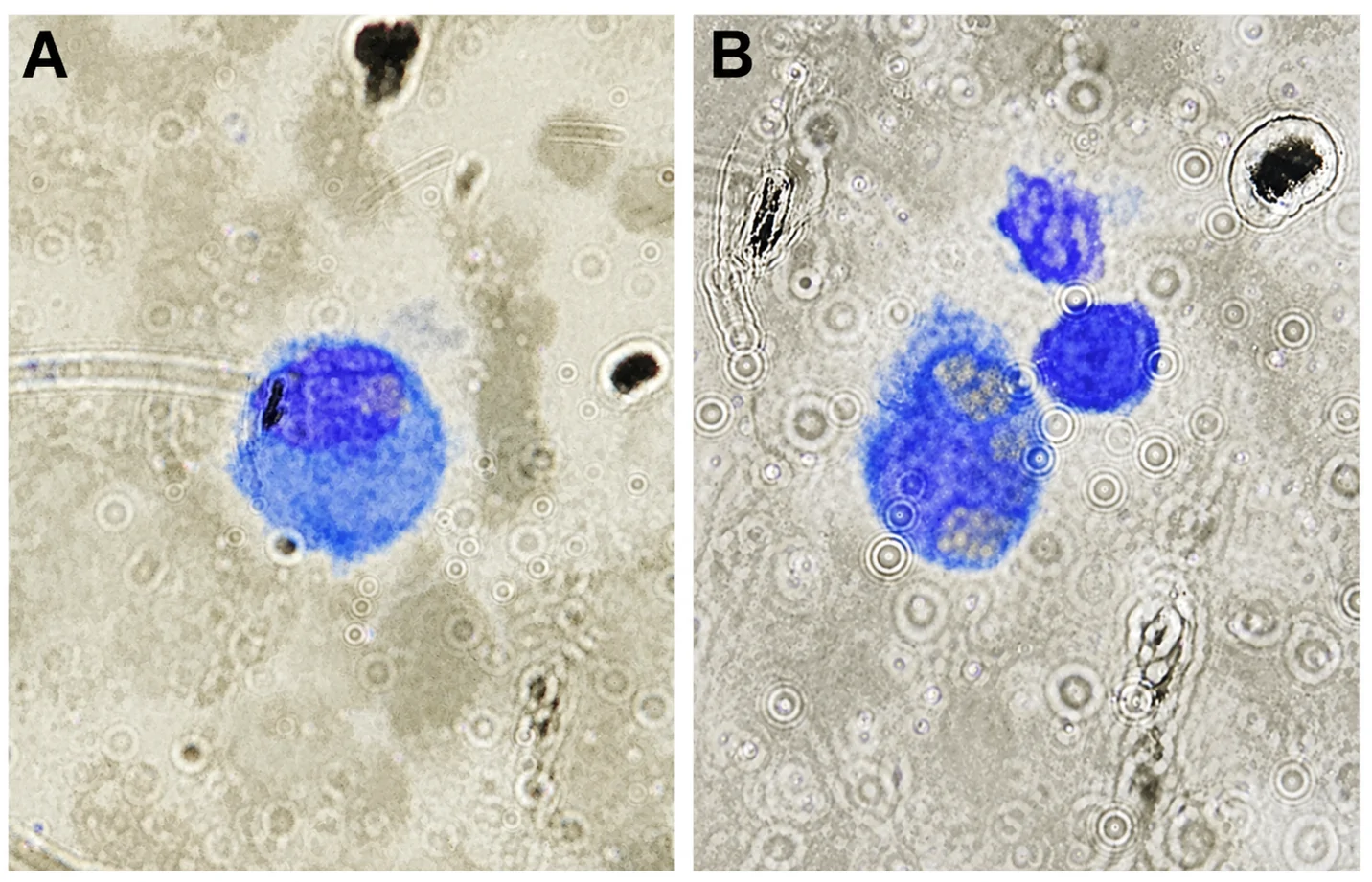
Bone marrow aspirate showing hemophagocytosis: (**A**) Large histiocytic cell with abundant pale basophilic cytoplasm containing phagocytosed hematopoietic elements, consistent with hemophagocytosis. (**B**) Bone marrow aspirate field showing a macrophage-like cell with cytoplasmic vacuolization and engulfed cellular material, supporting marrow hemophagocytosis in the setting of secondary HLH. Both images were acquired using a 100× objective.

**Figure 2:**
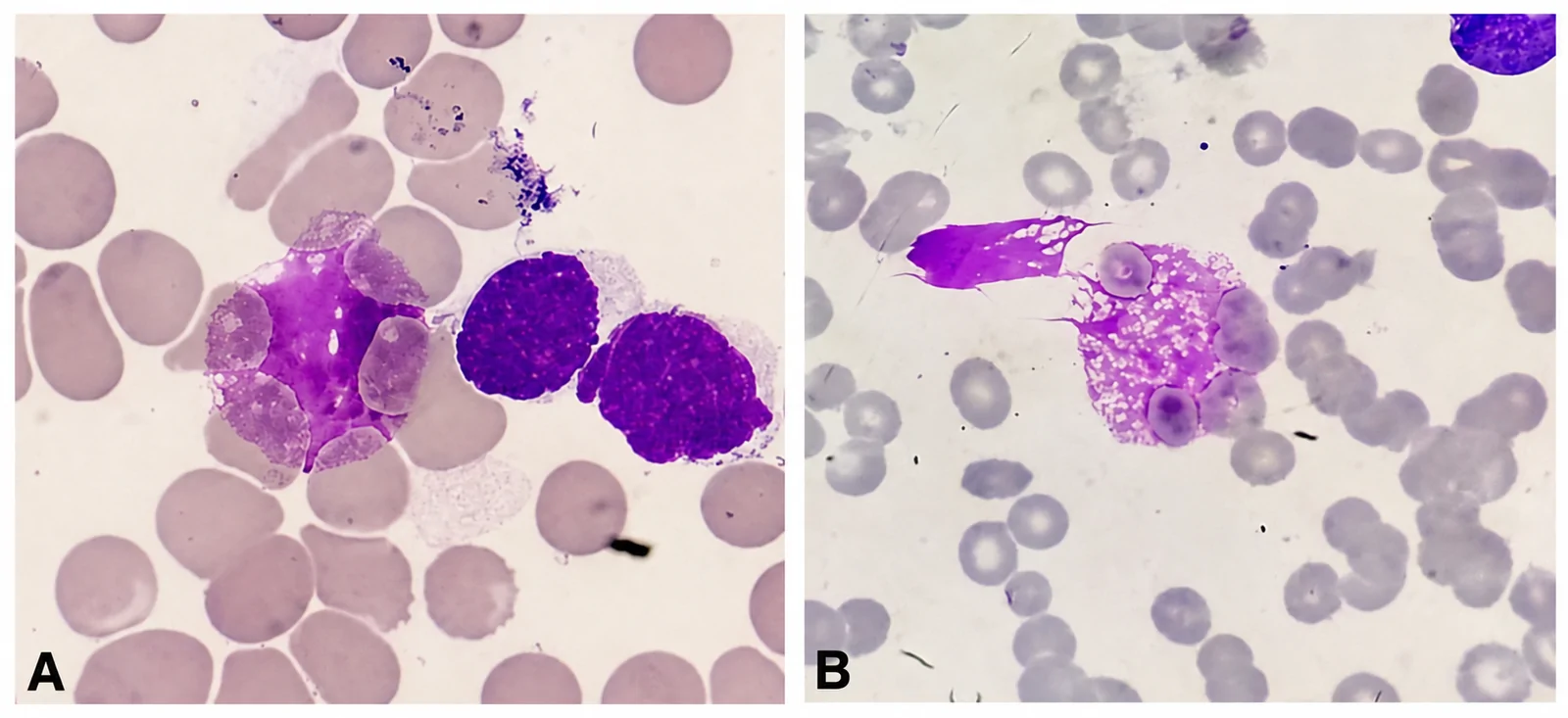
Bone marrow aspirate showing hemophagocytosis in secondary HLH associated with disseminated histoplasmosis: (**A**), Large histiocytic cell with abundant pale basophilic cytoplasm containing phagocytosed hematopoietic elements, consistent with hemophagocytosis. (**B**), Bone marrow aspirate field showing a macrophage-like cell with cytoplasmic vacuolization and engulfed cellular material, supporting marrow hemophagocytosis in the setting of secondary HLH.Original magnification: (**A**), 100×; (**B**), 40×.

**Figure 3:**
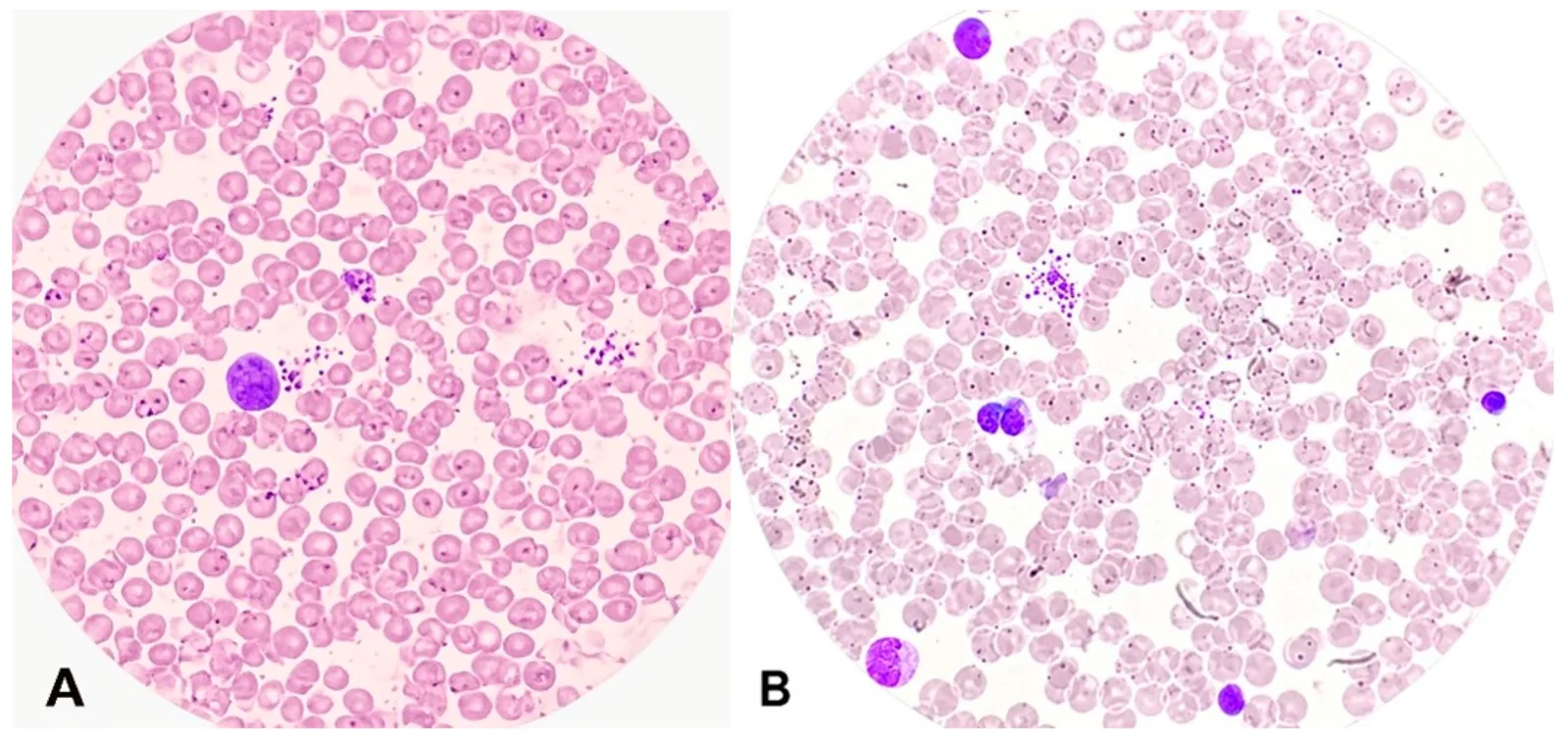
Bone marrow aspirate findings in the patient with visceral leishmaniasis-associated secondary HLH: (**A**,**B**) Representative bone marrow aspirate fields containing amastigote-like forms morphologically compatible with *Leishmania* spp. The formal bone marrow aspirate report did not demonstrate hemophagocytosis, and bone marrow PCR for *Leishmania* spp. was negative. The diagnosis of visceral leishmaniasis was supported by the epidemiological context, a positive rapid diagnostic test, and reactive indirect immunofluorescence (1:256). Both images were acquired using a 40× objective.

**Table 1:** Characteristics of the patients.

Variable	Case 1	Case 2	Case 3
Age, sex	54, male	34, male	28, male
Underlying condition	End-stage kidney disease on hemodialysis	Advanced HIV infection (stage C3)	Previously healthy
Initial clinical impression	High-risk febrile neutropenia/sepsis	Opportunistic infection/sepsis	Prolonged febrile syndrome/presumed infection
Main trigger associated with HLH	Acute myeloid leukemia	Disseminated histoplasmosis	Visceral leishmaniasis
Persistent fever	Yes	Yes	Yes
Cytopenias	Pancytopenia	Bicytopenia	Pancytopenia
Organomegaly	Splenomegaly	Hepatosplenomegaly	Hepatosplenomegaly
Ferritin (ng/mL)	8975	3551	8856
Triglycerides (mg/dL)	789	284–347	230
Fibrinogen (mg/dL)	593	291	148 nadir; 202 at initial work-up
Bone marrow hemophagocytosis	Present	Present	Not demonstrated
HLH-2004 criteria fulfilled	5/8	7/8	4/8 initially; 5/8 during hospitalization
HScore	222	246	248 (retrospective)
Additional bone marrow findings	AML-M2 infiltration	Intracellular fungal structures	No neoplastic infiltration; normal 46,XY karyotype; amastigote-like forms reviewed clinically
Initial anti-infective management	Broad-spectrum antibiotics	TMP-SMX and broad antimicrobial coverage	Empiric antimicrobial therapy; prior antituberculosis therapy
Hyperinflammation-directed therapy	Supportive care; no etoposide-based regimen documented	Dexamethasone 4 mg intravenously every 12 h	Corticosteroids
Trigger-directed treatment	Azacitidine	Liposomal amphotericin B	Liposomal amphotericin B
Clinical outcome	Unfavorable	Clinical improvement; discharged with outpatient follow-up	Clinical and hematologic recovery

Note: The HScore for Case 3 (248) was calculated retrospectively using available pretreatment values obtained during the diagnostic work-up: temperature, 38.6 °C; hepatosplenomegaly; three-lineage cytopenia; ferritin, >6000 ng/mL; triglycerides, 230 mg/dL (~2.6 mmol/L); fibrinogen, 2.02 g/L; aspartate aminotransferase (AST), 30 U/L; no known immunosuppression; and no bone marrow hemophagocytosis.

**Table 2:** Overlapping and potentially discriminating features of HLH and sepsis.

Feature	HLH	Sepsis	Clinical Interpretation
Ferritin	Often markedly elevated and may rise rapidly	May also be markedly elevated in severe infection or organ injury	Marked hyperferritinemia is a red flag but is not a standalone discriminator
Cytopenias	Often progressive and multilineage	Variable; may occur in severe sepsis or DIC	Progressive multilineage cytopenias should increase suspicion for HLH
Splenomegaly	Common	Less typical	Supports HLH when present but is not specific and may reflect the underlying trigger
Triglycerides/fibrinogen	Hypertriglyceridemia and/or decreasing fibrinogen are characteristic	Abnormalities may occur, particularly in sepsis complicated by DIC	Serial trends and combinations of abnormalities are more informative than isolated values
CRP	May be elevated	Frequently elevated	CRP alone cannot reliably distinguish HLH from sepsis
Procalcitonin	Not part of HLH diagnostic criteria; may be influenced by concomitant infection	Often elevated in bacterial sepsis	Elevated PCT supports bacterial infection but does not exclude concomitant HLH
Bone marrow hemophagocytosis	May be present or absent	May also occur in severe infection or critical illness	Supportive when present but neither necessary nor sufficient for the diagnosis of HLH
Response to appropriate antimicrobial therapy	Persistent or disproportionate inflammation should prompt reassessment	Clinical improvement is generally expected when infection is adequately controlled, although trajectories vary	Failure to improve as expected should prompt parallel evaluation for HLH and other causes

**Table 3:** Practical red-flag pathway for adults with presumed sepsis who are not improving.

Step	Practical Action
1. Continue sepsis care	Do not delay appropriate cultures, source control, or antimicrobial therapy while evaluating for HLH.
2. Reassess the inflammatory trajectory	Increase suspicion when fever, inflammation, or organ dysfunction persists or worsens despite appropriate treatment.
3. Identify HLH red flags	Look for progressive multilineage cytopenias, splenomegaly, markedly elevated or rising ferritin levels, hypertriglyceridemia, decreasing fibrinogen, and otherwise unexplained hepatic injury or coagulopathy.
4. Quantify HLH probability	Calculate the HScore and assess HLH-2004 criteria using serial clinical and laboratory data. Do not wait for bone marrow hemophagocytosis before pursuing the diagnosis.
5. Search for the trigger in parallel	Evaluate for infection, hematologic malignancy, autoimmune or autoinflammatory disease, and drug- or immune-mediated causes according to the clinical context.
6. Individualize treatment	Treat the underlying trigger promptly and individualize corticosteroid, etoposide, or other immunomodulatory therapy according to disease severity, trigger, organ dysfunction, comorbidities, and response to initial treatment. Involve hematology and other relevant specialties early.

## Data Availability

The original contributions presented in this study are included in the article. Further inquiries can be directed to the corresponding author.

## Abbreviations

HLH	Hemophagocytic lymphohistiocytosis
AML	Acute myeloid leukemia
HIV	Human immunodeficiency virus
NK	Natural killer
CRP	C-reactive protein
PCT	Procalcitonin
DIC	Disseminated intravascular coagulation
PCR	Polymerase chain reaction
TMP-SMX	Trimethoprim-sulfamethoxazole
